# Measuring affective symptoms of depression in aphasia: development of an accessible ecological momentary assessment tool

**DOI:** 10.1007/s11136-026-04292-y

**Published:** 2026-06-06

**Authors:** Brooke Boxrud, Eleanor Siegle, Stewart A. Shankman, Madhu Reddy, James W. Griffith, Sameer A. Ashaie

**Affiliations:** 1 Think and Speak Lab, Shirley Ryan AbilityLab, Chicago, IL USA; 2 Stahl Center for Psychiatric Neuroscience, Department of Psychiatry and Behavioral Sciences, Northwestern University, Chicago, IL USA; 3 Department of Informatics, University of California, Irvine, Irvine, CA USA; 4 Obstetrics and Gynecology, University of Chicago, Chicago, IL USA; 5 Roxelyn and Richard Pepper Department of Communication Sciences and Disorders, Northwestern University, Evanston, IL USA; 6 Department of Physical Medicine and Rehabilitation, Feinberg School of Medicine, Northwestern University, Chicago, IL USA

**Keywords:** Aphasia, Depression, EMA, PPI, Focus group, Cognitive interview

## Abstract

**Purpose:**

Post-stroke depression is highly prevalent in aphasia, yet existing depression measures rely heavily on language and lack sufficient validity for this population. The aim of this study was to develop an aphasia-accessible Ecological Momentary Assessment (EMA) of depression based on the input of people with aphasia, their care partners, and speech-language pathologists (SLPs).

**Methods:**

Nine focus groups were conducted with people with aphasia (*n* = 15), care partners (*n* = 13), and SLPs (*n* = 13) to identify relevant depression symptoms. Items were selected based on factors such as endorsement ratings and qualitative feedback across stakeholder groups. Participants with aphasia also took part in individual cognitive interviews to ensure comprehensibility and accessibility of the final items, corresponding pictures, and pictorial rating scale.

**Results:**

The final set of items to be included in the EMA consisted of three positive affect items (*determined*, *proud*, *interested*) and three negative affect items (*sad*, *like a failure*,* angry*). Cognitive interviews confirmed comprehensibility and accessibility of the items, though the picture for *interested* required revision. Additionally, participants found 3–4 daily assessments feasible.

**Conclusion:**

Stakeholder engagement revealed that positive affect dysregulation (e.g., reduced interest in previously rewarding activities) may be particularly salient for depression in aphasia in addition to negative affect dysregulation (e.g., increased feelings of failure and anger). The resulting six-item EMA uses multimodal supports (e.g., text, pictures, pictorial rating scale, audio recordings) to capture both valence systems.

**Supplementary Information:**

The online version contains supplementary material available at 10.1007/s11136-026-04292-y.

## Abstract (plain language summary)

Existing depression measures rely heavily on language and lack sufficient validity for people with post-stroke aphasia. Our aim was to develop an aphasia-accessible measure of depression based on the input of people with aphasia, their care partners, and speech-language pathologists (SLPs). We conducted focus groups for people with aphasia, their care partners, and SLPs to identify relevant depression symptoms. The process of selecting items to include in the final measure considered both endorsement ratings and participant feedback from focus groups. Participants with aphasia also took part in individual cognitive interviews to ensure comprehensibility and accessibility of the final items, corresponding item pictures, and a pictorial rating scale to rate their mood. The final items included in our measure consisted of three positive emotions (*determined*, *proud*, *interested*) and three negative emotions (*sad*, *like a failure*,* angry*), as participant engagement revealed that dysregulation of positive emotions (e.g., reduced interest in previously rewarding activities) may be particularly salient for depression in aphasia in addition to dysregulation of negative emotions (e.g., increased feelings of failure and anger).The final measure of depression uses multimodal supports (e.g., text, pictures, pictorial rating scale, audio recording).

**Supplementary Information:**

The online version contains supplementary material available at 10.1007/s11136-026-04292-y.

## Introduction

Depression impacts around 30% of people with stroke and is associated with longer hospital stays, increased mortality rates, and an overall worse quality of life [1–3]. Post-stroke depression is highly prevalent in people with aphasia, a multimodal disturbance of language (e.g., reading, writing, speaking, and understanding) that impacts one-third of stroke survivors [4, 5]. This disturbance in language makes assessing depression in these individuals challenging as current depression assessments rely heavily on language. Moreover, current depression assessments are not typically validated for people with aphasia and do not take their experiences into account [6–8]. Understanding how depression impacts people with aphasia is essential to improving their quality of life, and this process begins with constructing valid assessment tools that consider their perspectives and those that advocate for them [9]. Given the unique communication challenges faced by people with aphasia, traditional assessment approaches may not adequately capture depression in this population, emphasizing the need for improved measures.

To address these challenges and improve the validity and reliability of depression and quality of life measures, Patient-Reported Outcome Measures (PROMs) have become increasingly important. PROMs are assessments that capture information directly from patients about their symptoms, functioning, and quality of life, aiding in the provision of person-centered care [10, 11]. Furthermore, Patient and Public Involvement (PPI) in the development of PROMs has been shown to increase their relevance to users, identify aspects of health not previously considered by researchers, and improve overall quality of research [9, 12]. PPI in aphasia research ensures people with aphasia and other relevant stakeholders (e.g., care partners and speech-language pathologists) are represented throughout the research process and that the research is ethical, responsible, and relevant [13]. Appropriate PROMs are generally considered to have PPI throughout their development, yet many PROMs in use fail to report how patients and other relevant stakeholders were involved in the research process [14, 15]. For example, a recent systematic review of PROMs in communication disorders, including aphasia, rated 21 out of 25 PROMs as inadequate due to a lack of clear evidence of PPI in their development, raising concerns about the validity of the measures currently in use [16, 17].

A related challenge is that existing depression measures for individuals with aphasia often attempt to assess a heterogeneous list of symptoms from multiple domains of depression (e.g., cognitive, affective, and somatic). This may pose a particular challenge for some people with aphasia who, due to deficits in executive functioning, may struggle with shifting between thinking about these different domains of symptoms and behaviors [18]. Additionally, somatic symptoms (e.g., fatigue, psychomotor agitation or retardation, diminished ability to think or concentrate, and sleep disturbances) may be due to the neurological impact and/or physical impairments of stroke rather than recognized as indicators of depression [19–21].

An alternative approach is to focus only on one domain of depression. The *affective* domain of depression, specifically the dysregulation of negative affect features (e.g., increased sadness, feelings of failure, and anger) and positive affect features (e.g., decreased pride, motivation, and interest in previously rewarding activities), provides an especially effective framework for evaluating symptoms of depression in people with aphasia. Furthermore, these are core features in numerous theoretical models of depression, such as the tripartite model of depression, and often drive the disorder’s impairment in functioning [22–25].

Present depression measures for people with aphasia are limited as they often rely on care partner reports and may miss unobservable internal emotional states [26]. These measures also often fail to capture dysregulation in both positive and negative affect [7, 27]. This is a notable omission, as communication impairments can diminish autonomy and restrict participation in goal-directed and socially rewarding activities, which are core correlates of positive affect dysfunction. Ecological Momentary Assessments (EMAs) offer an alternative approach, serving as a well-suited methodological framework for capturing day-to-day symptom variability in real-world environments [28].

**Fig. 1:**
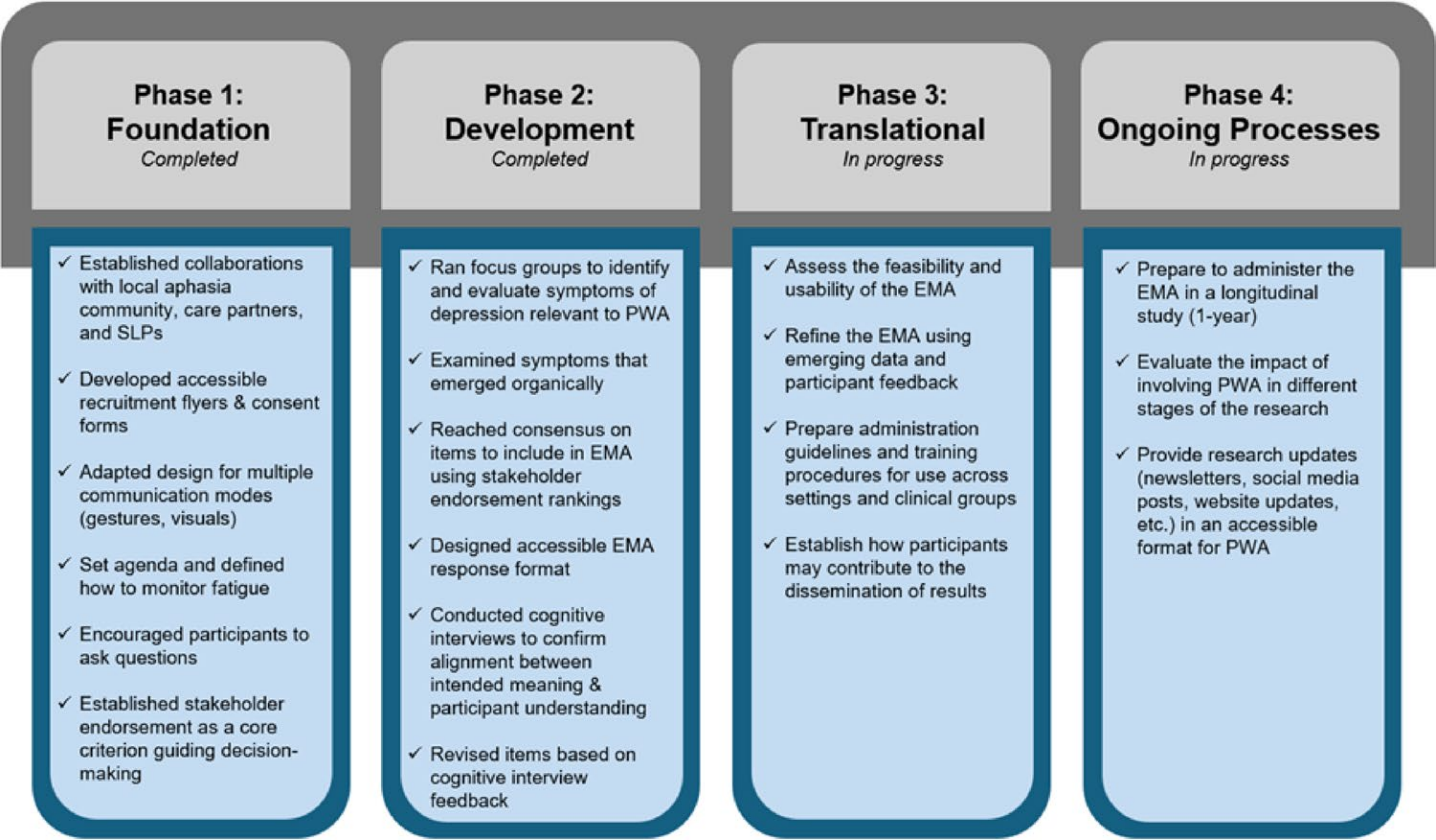
Situating our approach to PPI within the PAOLI framework

The aim of the present study was to develop an EMA that effectively captures daily fluctuations in both positive and negative affective symptoms of depression in people with aphasia. EMA methods have been shown to be feasible for assessing affect, life satisfaction, and symptoms (e.g., depression, pain, and fatigue) in populations such as people with dementia and stroke survivors without aphasia [29–32]. EMA approaches have also been used in aphasia research, providing evidence that these methods can effectively capture moment-to-moment variations in functioning and lived experience [33]. To develop this measure, we first conducted separate focus groups consisting of people with aphasia, their care partners, and speech-language pathologists (SLPs) to identify which depression symptoms are most important to them. Individual cognitive interviews were also conducted for participants with aphasia to assess comprehensibility and accessibility of proposed EMA items [34, 35]. PPI was a guiding principle throughout the development process to ensure the EMA’s validity, accessibility, and alignment with the lived experiences of people with aphasia, and we situated our approach to PPI within the People with Aphasia and Other Layperson Involvement (PAOLI) framework [9, 36–38]. See Fig. 1 for a visual overview of how we situated our approach to PPI within the PAOLI framework.

## Methods

### Participants

Separate focus groups were conducted with people with aphasia (*n* = 15), their care partners (*n* = 13), and speech-language pathologists (SLPs, *n* = 13) to evaluate the perceived importance of various depression symptoms and to determine which symptoms are most salient for people with aphasia. Participants with aphasia also completed individual cognitive interviews after participating in the focus groups. Participants were recruited from the community through phone calls and e-mails.

All participants with aphasia were English speakers with chronic left-hemisphere stroke and no comorbid neurological conditions affecting their communication. Participants’ aphasia severity ranged from severe to mild on the Aphasia Quotient of the Western Aphasia Battery-Revised [39]. Thirteen out of the 15 participants with aphasia met criteria for major or minor depression according to the Center for Depression Epidemiologic Scale [40]. Care partners in this study had been caring for people with aphasia for a range of 1 to 14 years, with an average of 5.76 years, and there were several different care partner relationships, including spouses (*n* = 9), parents (*n* = 2), an adult child (*n* = 1), and a sibling (*n* = 1). Years of experience among SLPs working with people with aphasia varied, with an average of 13.38 years. Table 1 summarizes participant demographics. This study was approved by the Northwestern University Institutional Review Board (IRB#STU00217329). All participants provided written consent prior to enrollment in the study.

**Table 1:** Demographics of people with aphasia, care partners, and speech-language pathologists

Participant	Age	Gender	Race	TPO (years)	Relationship to PWA	Time spent caring (years)	Years working with PWA	CESD	AQ
PWA01	54	M	White	6				8	72.1
PWA02	49	M	White	9				22	94.2
PWA03	58	F	Black	3				21	93.2
PWA04	31	F	White	5				32	80
PWA05	62	M	–	6				8	54.5
PWA06	46	F	Asian	4				18	77.7
PWA07	54	F	White	2				10	63.3
PWA08	70	F	White	10				17	66.2
PWA09	39	M	Black	4				12	71.6
PWA10	58	M	White	1				27	45.5
PWA11	44	M	White	1				25	69.8
PWA12	56	M	White	14				4	58.9
PWA13	67	M	White	1				31	53.8
PWA14	43	F	White	17				23	88.6
PWA15	65	M	White	11				3	34.7
CP01	55	F	–		Spouse	6			
CP02	48	F	Asian		Spouse	9			
CP03	70	M	White		Parent	5			
CP04	59	F	–		Spouse	6			
CP05	35	F	Black		Child	3			
CP06	52	M	White		Spouse	3			
CP07	74	M	White		Spouse	10			
CP08	59	F	Black		Sister	4			
CP09	43	M	White		Spouse	1			
CP10	43	F	White		Spouse	2			
CP11	77	F	White		Parent	14			
CP12	67	F	White		Spouse	1			
CP13	60	M	White		Spouse	11			
SLP01	30	F	White				7		
SLP02	30	F	White				6		
SLP03	33	F	White				10		
SLP04	35	F	White				11		
SLP05	28	F	White				4		
SLP06	35	F	White				11		
SLP07	30	F	White				2		
SLP08	67	F	White				43		
SLP09	55	F	White				30		
SLP10	32	F	White				9		
SLP11	36	F	White				11		
SLP12	41	F	White				18		
SLP13	35	F	Black				12		

PWA = person with aphasia, CP = care partner, SLP = speech language pathologist, TPO = time post onset (years), CESD = Center for Epidemiologic Studies Depression Scale, AQ = aphasia quotient of the western aphasia battery-revised. A dash indicates no response given.

Table 1 goes here.

**Fig. 2:**
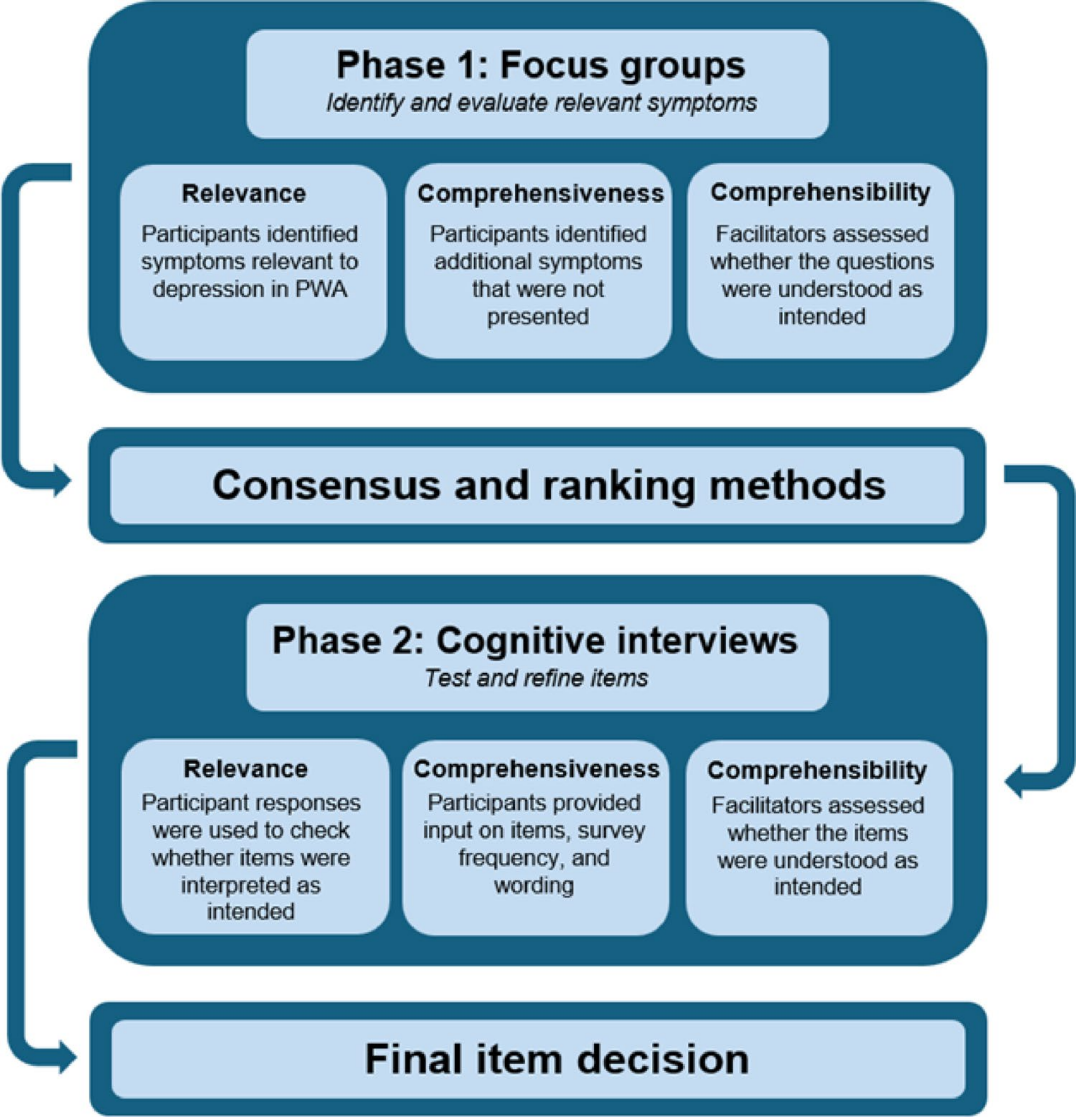
Reporting content validity following COSMIN guidance

To ensure strong content validity, we followed COSMIN guidance to develop our items by systematically evaluating the relevance, comprehensiveness, and comprehensibility of all candidate items through the focus groups and cognitive interviews [41, 42]. See Fig. 2 for a visual overview of how COSMIN guidance was applied throughout the research process. Additionally, see supplementary materials for a completed COREQ (Consolidated criteria for Reporting Qualitative research) Checklist for a report on our research team, study methods, context of the study, findings, analysis, and interpretations [43].

## Phase 1: focus groups

Nine focus groups were conducted over Zoom: three with people with aphasia, three with care partners, and three with SLPs. Each group contained four or five participants and took between two and three hours to complete. A total of three facilitators participated in the study (one SLP, one post-doctoral fellow, and a research assistant), with two present at each focus group. Several participants were familiar with the facilitators through their longstanding involvement in the aphasia community. A semi-structured interview guide was developed and implemented in the focus groups to (1) elicit discussion on participants’ general experiences with depression and (2) elicit discussion on the relative importance of 28 different affective items from the Positive and Negative Affect Schedule (PANAS) and the Patient-Reported Outcomes Measurement Information System (PROMIS) Emotional Distress – Depression – Short Form [44–46]. We treated each question from the two scales as unique items for participants to give their input on. We focused on items from these scales given their widespread use in assessing negative mood (e.g., sadness), views of self (e.g., worthlessness), and social cognition (e.g., loneliness). Additionally, these measures assess decreased positive affect (e.g., loss of interest) [47]. See supplementary materials for complete focus group guide.

## Focus groups: relevance

In groups consisting of people with aphasia, participants were asked whether the presented item was relevant to their experience of depression and prompted to provide a yes/no response. Some participants with aphasia explained their reasoning for endorsing or rejecting items, and facilitators asked probing questions about each item’s importance to experiencing depression. Care partners and SLPs were asked similar questions to elucidate the importance of different symptoms. Discussions naturally evolved as participants elaborated on why some items were more pertinent to their experiences with depression than others. Additionally, all participants were shown examples of response scales (e.g., slider scale) and asked to evaluate the accessibility and suitability of each scale. See supplementary materials for examples of response scales shown during the focus groups.

## Focus groups: comprehensiveness

Participants were encouraged to identify any additional symptoms of depression that were not included in the provided list of items. This allowed us to determine whether the candidate items captured the full range of affective experiences associated with depression for people with aphasia.

## Focus groups: comprehensibility

Each item was presented one at a time for people with aphasia using pictorial supports and aphasia-accessible communication strategies, alternating between items with negative affect and items with positive affect to reduce priming effects. For care partners and SLPs, 3 or 4 items were presented at a time since additional supports were not needed for the discussion. Facilitators asked clarifying questions when needed and took notes on items that required explanation to inform the refinement process. To ensure that endorsements genuinely reflected participants’ experiences of depression, facilitators used brief probes to clarify how participants were interpreting each symptom. Participants repeatedly linked certain items to their depression, which strengthened confidence that the items reflected depressive experiences rather than general mood.

After each focus group, we compiled endorsements for the items, including any organic (unprompted) mentions. We then ranked the items based on the total number of endorsements per group as well as participant feedback. These consensus and ranking methods allowed us to assess participant agreement on issues lacking consensus, quantify qualitative data, and identify items to include in phase 2: cognitive interviews [48].

### Phase 2: cognitive interviews

Cognitive interviewing is a method for exploring how people make sense of survey questions, aiming to improve clarity and usability [34, 49]. Through this method, we advanced the development of the EMA by integrating feedback from participants with aphasia, helping ensure it will be accessible to those for whom it is designed.

## Cognitive interviews: relevance

The same participants with aphasia who took part in the focus groups took part in individual cognitive interviews. Their responses were used to check whether the proposed EMA items, including the wording, visuals, and the chosen pictorial rating scale, were understood as intended.

## Cognitive interviews: comprehensiveness

Researchers ES and BB met with participants over Zoom, with ES conducting the cognitive interviews in the first half of the meeting and BB administering the WAB-AQ in the second half. The interview protocol was structured by item, and participants were asked additional probing questions to elicit deeper responses before proceeding to the next item. For each of the proposed EMA items, participants were first asked about their initial impressions of the visuals that were developed (i.e., “what emotion do you see in this picture?"). After providing their initial interpretations, participants were asked if they thought the image accurately depicted the item (i.e., “do you think this picture shows SAD?"). Additional questions were asked to delve deeper into participants’ interpretations, including (a) how they answered certain questions using the pictorial rating scale (e.g., “if you were feeling SAD right now, how would you rate your mood on this scale?"), (b) how they defined certain words (e.g., “what does SAD mean to you?", “what does RIGHT NOW mean to you?"), (c) what time period they considered when answering the questions (e.g., “were you thinking about this week, last week, earlier today, or right now?”), and (d) how difficult the questions felt (e.g., “was this question easy, medium, or hard to understand?”). At the end of each interview, participants were asked to indicate how many times per day they would be willing to rate their moods using the proposed item pictures and pictorial rating scale, with five times per day suggested as a starting point.

### Cognitive interviews: comprehensibility

To ensure full participation by people with aphasia, ES and BB used supported communication strategies in the cognitive interviews (e.g., clear wording, slower pacing, written key words, and visual cues). Participants were encouraged to respond using modalities they were comfortable with, and facilitators checked understanding by summarizing and confirming key points. When language difficulties arose, facilitators rephrased questions, offered choices, or provided cues to help participants express their ideas. Fatigue was monitored throughout each session through observation and brief check-ins. Breaks were offered when needed, and any adjustments were documented to ensure that participants’ input reflected their true views on depression symptoms rather than the effects of cognitive strain. To note, some questions were skipped for some participants due to time constraints and fatigue. See supplementary materials for complete cognitive interview guide.

### Analysis

#### Focus groups

Focus group recordings were saved, and their Zoom-generated transcripts were manually reviewed and corrected line-by-line to ensure accuracy. The final transcripts were de-identified and imported into Dedoose 9.2, a cloud-based qualitative coding software. Three researchers independently analyzed all focus group transcripts and documented every instance in which participants identified a symptom as relevant to the depressive experiences of people with aphasia. Following independent analysis, the researchers met to ensure consistency across the codes and endorsement rates. Codes were developed to capture participants’ reasoning for considering items as relevant or irrelevant to depression in people with aphasia. Researchers considered a symptom to be endorsed as relevant when participants signaled agreement either verbally or through clear nonverbal cues, such as nodding, thumbs up, or facial expressions indicating recognition or resonance with that symptom. Endorsement rates for each symptom were tallied across all nine focus groups. Researchers also analyzed participant input on the different types of response scales that may be optimal for people with aphasia, following the same steps for consensus coding.

After independent review, the researchers then compared their evaluations and conferred with the PI through discussion. Stakeholder endorsement directly guided item selection, and additional reviewers were available when disagreements persisted. Researcher input was incorporated only after all stakeholder feedback had been fully reviewed and integrated, ensuring that the final items reflected both participant perspectives and a transparent, systematic decision process.

### Cognitive interviews

Cognitive interviews were conducted via Zoom and lasted between one and two hours. The same steps for consensus coding used to analyze the focus group transcripts were applied to the cognitive interview transcripts. After coding was completed, the codes were synthesized into a table organizing participant feedback and responses to the proposed items. SAA and SAS met to assess participant feedback and document confusion, negative reactions, and/or misinterpretations that warranted revision or rejection of items. The researchers decided which items should be retained, revised, or rejected, and evaluated if the items altogether fulfilled the criteria of a PROM [42].

## Results

The following selection criteria were established for including an item in the final EMA: (1) item must be endorsed by at least 10 participants across a minimum of two of the three stakeholder groups, (2) item must be aphasia-accessible and feasible to depict visually, (3) final item set must represent both positive and negative affect symptoms, and (4) final item set must minimize participant burden to allow for intra-day assessments.

### Focus groups: items

Applying the selection criterion and qualitative insights from focus group discussions, five items met all selection criteria: *sad* (people with aphasia: 9/15, care partners: 13/13, SLPs: 13/13), *proud* (people with aphasia: 14/15, care partners: 13/13, SLPs: 10/13), *determined* (people with aphasia: 14/15, care partners: 13/13, SLPs: 13/13), *like a failure* (people with aphasia: 10/15, care partners: 12/13, SLPs: 11/13), and *interested* (people with aphasia: 14/15, care partners: 12/13, SLPs: 10/13). These items represent positive affect (*determined*,* proud*, and *interested*) and negative affect (*sad*,* like a failure)*, ensuring accessibility and minimizing conceptual redundancy (based on stakeholder comments). SAA discussed these items and the process of selecting them with co-authors SAS, JWG, and MR. All authors agreed with the final selection of items. These five items, along with their visual representations and the pictorial rating scale, were then evaluated through cognitive interviews (see below). Table 2 presents complete item endorsement data and qualitative insights for inclusion and exclusion decisions.

**Table 2:** Item endorsement from people with aphasia, care partners, and speech-language pathologists and inclusion/exclusion rationale

Item	PWA (*n* = 15)	CPs (*n* = 13)	SLPs (*n* = 13)	Decision and rationale
Positive affect items				
Determined	14	13	13	*Included* – High endorsement across groups. Stakeholders noted lack of determination feels like giving up; feels “bad”; important to ascertain motivation and agency.
Excited	15	13	12	*Excluded* – High endorsement across groups. Stakeholders noted that lack of excitement may be more to do with feeling of loss due to stroke.
Proud	14	13	10	*Included* – High endorsement across groups. The lack of feeling proud is related to feeling like a failure.
Enthusiastic	14	12	11	*Excluded* – Despite high endorsement across groups, stakeholders felt that the word may be difficult for some people with aphasia to understand.
Interested	14	12	10	*Included* – High endorsement across groups. Stakeholders opined that depression may reduce interest in usual activities and help-seeking.
Inspired	10	13	7	*Excluded* – Mixed endorsement across groups. SLPs thought that determined is more accessible than inspired, which feels too abstract.
Negative Affect Items				
Sad	9	13	13	*Included* – Despite low endorsement from PWA, stakeholders indicated the word is easily accessible and important for understanding mental health in PWAs.
Like a failure	10	12	11	*Included* – Consistent stakeholder endorsement. Linked to feeling sad, helpless, and the absence of things PWAs can do. Feeling like a failure is something PWAs often express to SLPs.
Angry/Frustrated^a^	11	13	13	*Included (angry)* – Emerged organically across stakeholder groups. Stakeholders felt that anger is key to understanding depression in PWAs while frustration is associated specifically with communicative abilities.
Depressed	11	11	9	*Excluded* – Despite endorsement across groups, stakeholders felt that *depressed* is too broad a concept; PWAs may not want to admit feeling depressed because of stigma.
Worthless	10	10	11	*Excluded* – Stakeholders linked it to depression: absence of “paycheck”. Conceptually redundant to feeling like a failure.
Hopeless	3	12	13	*Excluded* – Discordant endorsement pattern. CPs disliked this item.
Helpless	4	12	9	*Excluded* – Low endorsement from PWAs. Stakeholders felt it could be triggering for some people.
Unhappy	3	13	8	*Excluded* – Stakeholders felt that *unhappy* is not an accessible word.
Having nothing to look forward to	5	11	7	*Excluded* – Stakeholders noted having nothing to look forward to being linked to depression, but it may not be accessible.
Upset	–	5	6	*Excluded* – Low endorsement across groups. CPs and SLPs did not like this word and felt it was not concrete.
Other Items				
Alert	14	7	7	*Excluded* – Despite high endorsement from PWAs, stakeholders felt that alert could be linked to having energy and being social. SLPs and CPs disliked the word.
Active	6	5	4	*Excluded* – Low endorsement across groups. Stakeholders agreed that it could be related to depression but not specific to stroke and aphasia.
Strong	6	10	9	*Excluded* – Low endorsement across groups. Stakeholders felt the word was too ambiguous as it could relate to mental or physical strength.
Distressed	–	5	5	*Excluded* – Low endorsement across groups. Stakeholders indicated that the word could be triggering for PWAs.
Attentive	–	5	5	*Excluded* – Low endorsement across groups. SLPs felt that the word is not concrete; could be a result of stroke/separate from depression.

PWAs = people with aphasia; CPs = care partners; SLPs = speech-language pathologists. A dash indicates item was not presented to stakeholder group.

^a^Item emerged organically during focus group discussions rather than from pre-selected PROMIS or PANAS

Table 2 goes here.

### Focus groups: response scale

Most participants across the groups did not prefer slider scales. Care partners and SLPs made it clear that selecting/clicking a choice is better than using slider scales for people with aphasia. Additionally, participants across groups disliked the combination of words, numbers, and pictures on the same scale as these scales can be “overwhelming” and “confusing”. Participants across groups preferred facial emojis as a response scale but felt that more than five faces were too many. Care partners did not prefer red as a color for facial emojis.

### Development of item pictures and pictorial rating scale

**Fig. 3:**
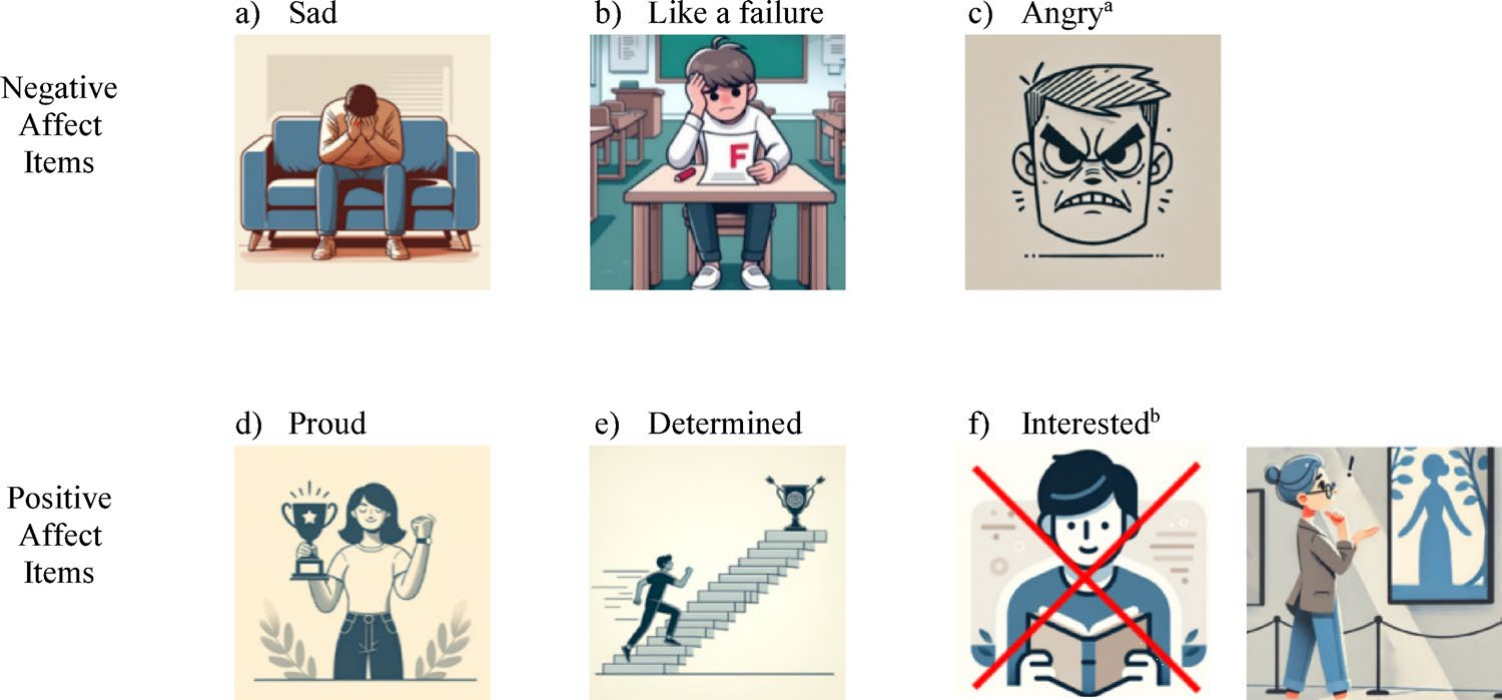
ChatGPT4-generated pictures for the final item set: **a** sad, **b** like a failure, **c** angry, **d** proud, **e** determined, and **f** interested. ^a^Angry was included upon further review. ^b^Interested was revised; the left image is crossed out to denote rejection, and the right image shows the updated version

**Fig. 4:**
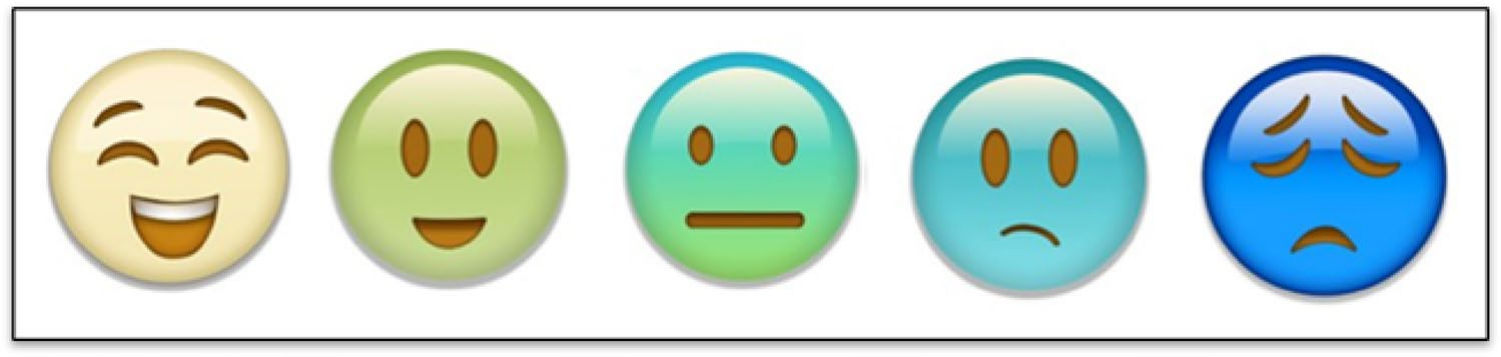
Pictorial rating scale

ChatGPT4 was used to develop pictures that depict each of the five items, and the researchers reached a consensus on the images that best represented the items (Fig. 3). To note, *angry* was selected upon further review (see subsequent section for the rationale behind the inclusion of *angry*). The software EmojiMaker was used to develop an aphasia-accessible pictorial rating scale, taking into consideration stakeholder input (Fig. 4). The generated pictures and scale were used in the cognitive interview portion of the study.

### Cognitive interviews

Overall, the pictures presented for each item were generally perceived by participants in accordance with our intended meaning (e.g., participants consistently described the picture for *sad* as “sad” without being prompted), with one exception: *interested* required revision (see subsequent section for the rationale behind the revision of *interested*). Overall, participants found the questions for each item to be easy to understand. Participants found familiar items like *sad* to be easy to understand, but more complex items like *interested* were harder to conceptualize. We present findings for the pictorial rating scale first, followed by results for each individual item. See supplementary materials for complete data collected during the cognitive interviews.

### Pictorial rating scale

Participants were asked to evaluate the scale in Fig. 4 (i.e., “what does this [scale] look like?“, “what do the faces represent?") early in the interview to minimize potential bias from seeing it used with multiple items. Most participants talked about the scale going from “happy” (first face, left side) to “sad” (fifth face, right side). One participant said, “first face looks like happy… second face is excited or something… middle face is whatever… another [fourth] face is a little sad.” Some participants described the scale by communicating how they were feeling at that moment. For example, one participant drew the third face and first face to indicate that sometimes he feels like the third face, but in that moment, he feels like the first face. Overall, participants understood that the scale was intended to capture a range of emotions with positive affect being on the left side of the scale and negative affect being on the right side of the scale.

### Sad

For each item presented, the interview sequence began by eliciting participants’ initial interpretations of the picture. When asked what emotion they saw for the picture depicting *sad*, most participants said “sad”. Some participants gave additional interpretations (e.g., “bad”, “down in the dumps”) or used gestures to describe the visual (e.g., covering their face with their hands, imitating crying). All participants agreed that the picture for *sad* depicted the feeling of being sad.

To ensure participants understood the item and the scale, participants were asked to rate their mood if they were feeling sad at that moment and if they were not feeling sad at that moment (e.g., “if you were feeling SAD right now, how would you rate your mood on this scale?”, “if you were NOT feeling SAD right now, how would you rate your mood on this scale?”). Participants chose the fourth and/or fifth face on the scale when asked how they would rate feeling sad at that moment and the first and/or second face when asked to demonstrate not feeling sad at that moment. Of the 15 participants, 13 said that the questions felt easy to understand and 2 said the questions were medium to understand.

### Proud

Participants gave a variety of interpretations when shown the picture for *proud* (e.g., “happy”, “accomplishment”, “aiming to”). Some participants focused on the specific aspects of the picture and gave responses like “trophy” and “star”. All participants agreed that the picture for *proud* depicted the feeling of being proud.

Participants selected the first and/or second face when asked to rate their mood if they were feeling proud at that moment. When asked to rate their mood if they were not feeling proud, participants primarily selected the third, fourth, or fifth face. Of the 15 participants, 11 were asked whether the questions were easy, medium, or hard to understand; all 11 said that the questions were easy to understand.

#### Determined

Participants provided a range of responses when shown the picture for *determined*, reflecting the different dimensions of feeling determined (e.g., “accomplishing a goal”, “motivated”, “striving for”). All participants agreed that the picture depicted the feeling of being determined. Most participants chose the second face when asked to rate their mood if they were feeling determined at that moment. When asked to rate their mood if they were not feeling determined at that moment, participants tended to select the third, fourth, or fifth face. Of the 15 participants, 12 were asked whether the questions were easy, medium, or hard to understand; eight said the questions were easy to understand, one indicated between medium and hard, two said medium, and one said hard.

### Like a failure

Participants also gave a variety of responses when shown the picture for *like a failure* (e.g., “sad”, “bad”, “flunk”). Some participants used gestures or visuals to describe the emotion (e.g., gave thumbs down, drew sad face). All participants agreed that the picture for *like a failure* depicted feeling like a failure. Most participants chose the fourth and/or the fifth face when asked to rate their mood if they were feeling like a failure at that moment. When asked to rate their mood if they were not feeling like a failure at that moment, most participants chose the first and/or the second face. Of the 15 participants, 13 were asked whether the questions were easy, medium, or hard to understand; 11 said the questions were easy to understand and two said the questions were medium to understand.

#### Interested

The picture for *interested* elicited the greatest number of responses that were less connected to *interested* and instead emphasized the literal features of the picture (e.g., “school”, “reading”, “book”). Moreover, the variations in the interpretations and feedback were influenced by participants’ unique experiences of having aphasia. For example, some participants brought up their preference for audiobooks because their aphasia makes it difficult to read. For them, the visual of someone reading a book did not convey the intended feeling of being interested. Despite the varied interpretations, all participants agreed that the visual was intended to depict the feeling of being interested, though many noted it did not resonate with their personal experience. Most participants chose the first and/or second face as their response when asked to rate their mood if they were feeling interested at that moment. When asked to rate their mood if they were not feeling interested at that moment, most participants chose the third or the fourth face. Of the 15 participants, ten were asked whether the questions were easy, medium, or hard to understand; five indicated easy, one indicated between medium and easy, three indicated medium, and one indicated between medium and hard.

#### Decision to retain, revise, reject, or add items

Based on these cognitive interview findings, researchers made final decisions about which items to retain, revise, or reject. Four out of the five items were retained without modification: *sad*, *proud*, *determined*, and *like a failure*. All participants agreed that the pictures depicted the items, with their initial interpretations closely matching the intended emotions. However, the picture for *interested* was revised, as many participants did not resonate with the picture and initially provided interpretations that diverged from the intended emotion. Therefore, the researchers chose a different picture for *interested* (Fig. 3).

Following the cognitive interviews, we did an additional review of both the focus group and cognitive interview data to determine whether any additional symptoms of depression had been organically raised, ensuring that no relevant items had been overlooked. During the focus groups, references to feeling angry and related terms (e.g., frustrated, mad, aggressive) emerged primarily in the context of communication challenges related to aphasia. As a result, the item was not advanced to cognitive interviewing like the other five items. A closer examination of the focus group transcripts and cognitive interview data, however, made it clear that these communication challenges and the emotions that arise with these challenges (i.e., anger), were deeply embedded in how the participants with aphasia experienced depression. This connection was too central to ignore, despite not undergoing the same iterative vetting process as the other items, and the decision was made to include *angry* as a sixth item. Additionally, *angry* was selected over related items because of its linguistic simplicity. Figure 3 includes the picture for *angry*. The final selection of items included ones with positive affect (*proud*,* determined*,* interested*) and negative affect (*sad*,* like a failure*,* angry*).

#### Survey wording and frequency

Participants were also asked to explain how they interpreted specific words used in certain questions. When asked what “right now” means to them, most participants talked about how they were feeling or what they were doing at that time. For example, one participant said “present…Zoom meeting”, referring to being in a Zoom meeting at that moment. Another participant used gestures to communicate what “right now” means, holding his hands very close together to represent a very small segment of time.

Toward the end of each cognitive interview, we asked participants how many times per day they would be willing to rate their moods using the proposed item pictures and pictorial rating scale, with five times per day suggested as a starting point. Four participants wanted to answer a mood survey fewer than five times, with three times being cited as the most common response. One participant said they would like to complete the surveys more than five times a day, saying that seven times a day is preferable. Another participant said they would prefer to do it once a week. Three participants shared that five times a day was feasible for them. Based on this feedback, the researchers met as a team and decided to reduce the number of surveys to four times a day, which balanced participant burden with the goal of capturing mood variability throughout the day and represented a compromise between the range of preferences (1–7 times per day).

### Final EMA items

**Fig. 5:**
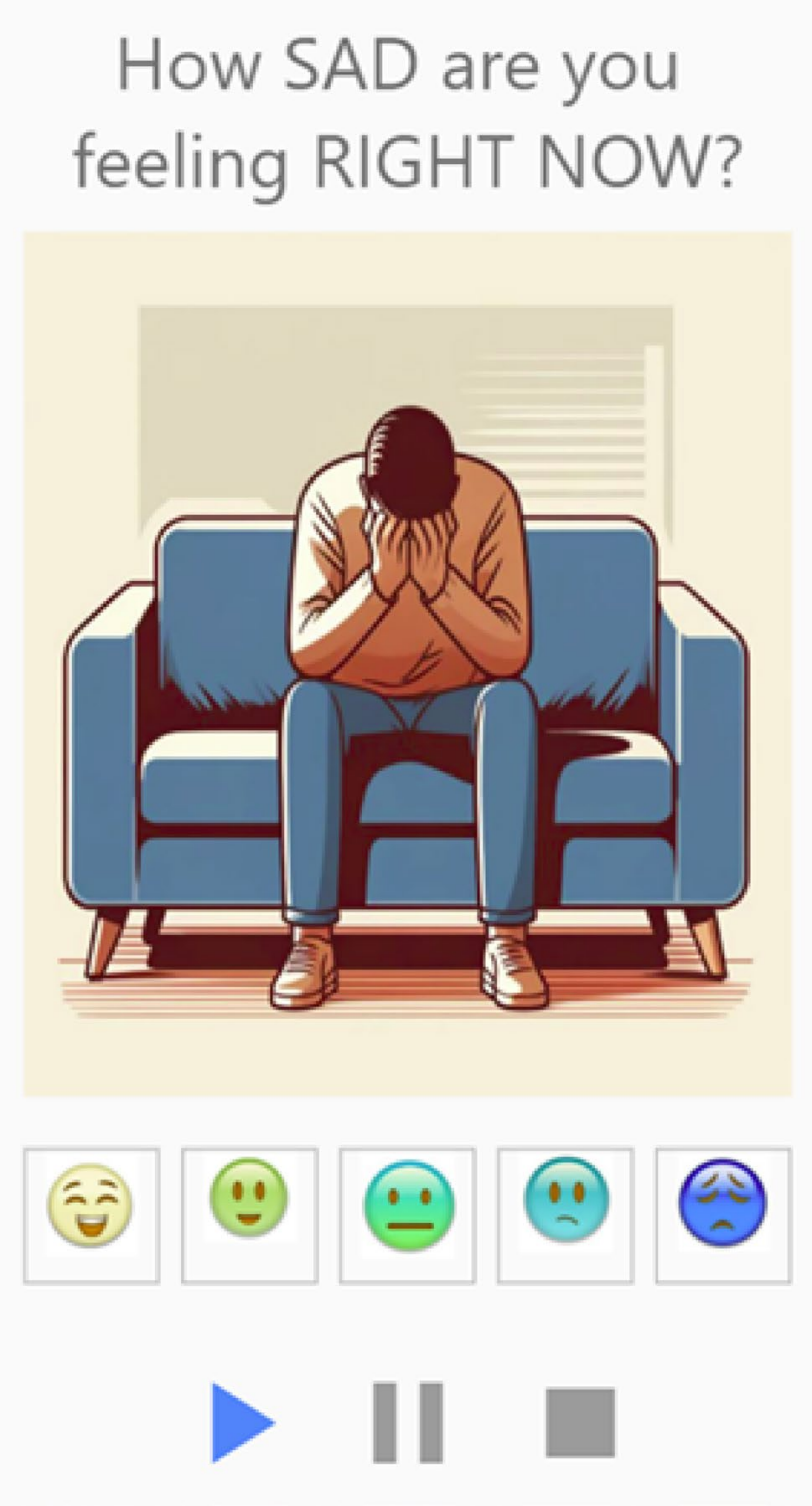
Example question in m-Path with multimodal supports (e.g., text, picture, pictorial rating scale, audio recording)

The final EMA includes six items (*interested*,* proud*,* determined*,* sad*,* like a failure*,* angry*). To ensure that our EMA remains aphasia-accessible, the final version being deployed to participants includes multimodal supports (e.g., text, pictures, pictorial rating scale, audio recordings). This ensures that participants can rely on different modalities for question interpretation. Figure 5 depicts how the question appears in m-Path, a smartphone-based EMA application [50]. The Flesch-Kincaid Grade Level for our EMA is 2.5, ensuring participants can comprehend the words [51].

## Discussion

Assessing depression in people with aphasia can be challenging since most current depression measures are language-based, inaccessible for people with aphasia, and developed without stakeholder input. To address these challenges, this study integrated Patient and Public Involvement (PPI) to create an aphasia-friendly tool, designed to measure both positive and negative affective dysregulation in depression. Given the importance of meaningful PPI in PROM development, we situated our approach to PPI within the People with Aphasia and Other Layperson Involvement (PAOLI) framework [38]. Our project incorporated PAOLI-informed approaches, such as establishing collaborations, adapting our design for multiple communication modes, and establishing stakeholder endorsements as core criterion to guide our decision-making. As we entered the development phase of the PAOLI framework, we ran focus groups to generate candidate EMA items based on participant experiences, designed an accessible EMA response format based on stakeholder feedback, conducted cognitive interviews to confirm many aspects of the EMA (e.g., affective depression symptom, corresponding picture, and question wording) were interpreted as intended, and revised EMA items based on participant feedback in cognitive interviews. See Fig. 1 for a visual depiction of how the PAOLI framework informed our processes and how we will incorporate PPI in future phases of our work.

In this study, people with aphasia and other relevant stakeholders (care partners, SLPs) endorsed the absence of positive emotions (e.g., *interested*) as central to depression, yet many traditional depression measures primarily focus on negative emotions and experiences. The endorsement of positive affect items alongside negative affect items suggests that people with aphasia experience depression not simply as heightened sadness or distress, but as a blunted response to positive affect fitting within multiple theoretical models of depression [22–25]. People with post-stroke aphasia may experience reduced autonomy due to linguistic impairment, hampering their engagement in goal-directed behavior (e.g., participation in therapy and social activities) [52, 53]. As a result, deficits in reward-based emotions may be a particularly salient marker of depression for people with aphasia.

Furthermore, focus group participants identified affective symptoms of depression that are often overlooked in standard assessments. Anger, for example, arose often and without prompt among participants. While anger is recognized as a depression symptom in some contexts, it rarely features prominently in common depression scales such as the PHQ-9 or aphasia-specific measures [54]. Anger is often conceptualized as an appetitive, approach-related emotion and has been strongly associated with depression [55, 56]. Anger may also be a more accessible and expressible emotion for people with aphasia compared to abstract concepts like hopelessness, making it a particularly valuable depression indicator in this population.

Cognitive interviewing has previously been shown to support validation of newly developed scales and can be applied to other relevant psychosocial issues faced by people with aphasia [57]. In this study, cognitive interviews were conducted for participants with aphasia to confirm that the selected items and pictorial rating scale were comprehensible. Items such as *sad*, *proud*, *determined*, and *like a failure* were consistently interpreted as intended. However, the original picture depicting *interested* (reading a book) was often associated with the participants’ aphasia-related deficits rather than the intended emotional state. This provides a concrete example of why generic visual representations are insufficient and why cognitive interviewing is essential for designing accessible PROMs. By ensuring accessibility through multimodal supports and an appropriate reading level in our EMA items, we have sought to minimize the confounding influence of language impairment on assessment, thereby aiming for a better measurement of the underlying constructs.

Additionally, current aphasia-specific tools to measure depression have different limitations. For example, the Stroke Aphasic Depression Questionnaire [26] relies on care partner observation rather than patient self-report, potentially missing internal emotional states that cannot be observed by others. Moreover, none of these measures were designed to capture deficits in both positive and negative affect that our stakeholder input revealed as central to depression in aphasia. By incorporating both patient self-report and multimodal accessibility features and using iterative cognitive testing and stakeholder input, our EMA addresses these gaps in aphasia depression assessment.

When asked about the feasibility of responding to EMA prompts multiple times per day, most participants preferred answering three times daily. Prior literature in aphasia research indicates four times a day is effective for high compliance in order to capture mood day-to-day [30]. Therefore, we decided to administer EMA four times a day. This feedback informed the final EMA design, balancing participant burden with the goal of capturing mood fluctuations over time.

## Clinical implications

These findings have implications for assessment and treatment of depression in people with aphasia. First, our EMA measure allows for repeated and ecologically valid measurements of depression symptoms in real-world contexts throughout the day, capturing mood variability that single-timepoint clinical assessments may miss. Second, by tracking both positive and negative affective symptoms, clinicians can identify individualized depression profiles that inform tailored interventions. Additionally, the EMA format can allow daily monitoring during treatment, allowing clinicians to track whether interventions are improving positive affect, reducing negative affect, or both. This is important since people with aphasia may have difficulty answering retrospective measures due to cognitive-communicative challenges. The multimodal supports also reduce the burden of language impairment on self-report, potentially yielding more accurate assessment than traditional language-heavy questionnaires.

### Limitations and future directions

Several limitations must be acknowledged. First, the item *angry* was included in the final EMA despite not undergoing the same cognitive testing as the other five items. Although participants appeared to understand the concept when discussed, we cannot be certain that the visual representation and item wording are optimally accessible. Its inclusion represents methodological limitation. Under COSMIN guidance, all items should be evaluated for relevance and comprehensibility with the target population, and future work should conduct dedicated cognitive interviews for this item.

Second, this study focused on EMA development and has not yet established psychometric properties. In the next phase, we will evaluate the measure’s reliability (e.g., internal consistency) and construct validity (convergent validity with existing depression measures). We will also explore EMA compliance and feasibility in people with aphasia.

Lastly, this study involved a relatively small group of chronic stroke survivors who were all English-speaking. Many participants were recruited from a similar community context. While this sample is appropriate for early-stage measure development, it does present limitations to the generalizability of the findings. Participant perspectives captured in this study may not fully represent individuals in different phases of recovery (e.g., acute, subacute) or people with different linguistic or cultural backgrounds.

In sum, this study demonstrates the indispensable value of stakeholder involvement and iterative cognitive testing in developing valid, person-centered outcome measures. Stakeholder involvement likely influenced the final set of EMA items by prioritizing symptoms that reflected lived experiences rather than those emphasized in clinical frameworks. While this may have altered the outcome compared to a purely researcher-driven process, it strengthened the validity and ensured our items were relevant to the population they are intended to serve. By grounding our tool in the lived experiences of people with aphasia, we have created an EMA assessment tool that captures symptoms associated with both positive and negative affective domains of depression.

## Supplementary Information

Below is the link to the electronic supplementary material.

Supplementary Material 1

Supplementary Material 2

Supplementary Material 3

## Data Availability

The data that support the findings of this study are available on request from the corresponding author. The data are not publicly available due to privacy or ethical restrictions.
