# Supplementary material for: Measuring affective symptoms of depression in aphasia: development of an accessible ecological momentary assessment tool
Source: Qual Life Res. 2026 Jun 6;35(7):175. doi: 10.1007/s11136-026-04292-y (PMC13242487; doi:10.1007/s11136-026-04292-y)
Supplement: Supplementary file 1 — Supplementary Material 1 [file 11136_2026_4292_MOESM1_ESM.docx]

**Focus Group Guide – Part 1**

“Mental health includes our emotional, psychological, and social well-being. It affects how we think, feel, and act. It also helps determine how we handle stress, relate to others, and make healthy choices”

**Q: How do you define mental health?**

**Q: Describe the importance of mental health in your life.**

*Additional probe: What makes those aspects important to you?*

*Additional probe: What does mental health mean to you? Has it changed after your stroke?*

**Q: Have you ever felt sad or depressed for an extended period of time?**

*Additional probe: How long have these experiences lasted? Months? Days? Weeks?*

*Additional probe: Has it been ongoing or do you experience it for short periods of time?*

*Additional probe: What do you think causes you to feel sad or depressed?*

*Additional probe: When you feel this way, can you describe your feelings or your thoughts?*

**Q: Depression can look different in different people and has many different features. What aspects of depression are the most important for your mental health?**

*Additional probe: Why are they important?*

*Additional probe: Can you tell me more about what you mean by [x]?*

**Q: Are there changes in your life because of depression?**

Additional probe: *Is there something specific to having aphasia that impacts this feeling?*

*Additional probe: Describe some of those changes.*

*Additional probe: Why did things change?*

*Additional probe: How have you coped with these difficult times?*

**For the next set of questions, participants were instructed to think about these questions in the context of the past 6 months.**

**Q: Have you recently experienced feelings of depression or sadness?**

*Additional probe: How have you known that you were depressed?*

*Additional probe: What caused you to feel depressed- Was there something specific to aphasia that affects your feelings?*

*Additional probe: How long did these feelings last?*

**Q: What words come to your minds when thinking about your own feelings and experiences of being depressed or sad?**

**Q: In the last six months, what emotions did you experience when you felt depressed?**

**Q: Were there any physical sensations that you felt when you were depressed?** {elicit examples – e.g., body aches, sleep disturbances, changes in appetite}

**Q: Were there any activities that you did or didn’t do when you feel depressed?**

**Q: What impact did you think depression had on your relationship with others?**

**Q: How did you describe your feelings of depression to your family or friends {elicit specific symptoms}**

**Q: Since your stroke, do you describe your feelings differently?**

**Q: How would you describe your feelings to a healthcare provider?**

*Additional probe: Do you describe these feelings differently to a family member or friend?*

**Q: What would be the most important things you would want to tell others if you feel depressed?**

**Q: How do you describe your feelings when you feel positive? What words come to you when describing your well-being?**

**Q: Is there anything we haven’t covered?**

**Focus Group Guide – Part 2**

*“Now, we are going to show some emotions and emotional states that we are interested in and how relevant they are to your mood, your mental health, and depression”*

For all items, the following questions were asked(Note, some questions for some items may have been skipped due to time constraints):

**Q: Would you say problems with *feeling [X]* is important to your overall mental health? To depression? Yes/No?**

*Additional probe: If this is important, tell me* ***why*** *it is important.*

**Q: Why do you think this emotion is/is not linked to mental health?**

**Q: Do you think this emotion is related to depression? Why?**

*Additional probe: tell me what makes this emotion linked to mood/mental health.*

PROMIS Emotional Distress—Depression—Short Form – 8 items

Positive and Negative Affect Schedule (PANAS-SF) – 20 items

1. Feeling *depressed?* [PROMIS]
2. Feeling *interested?* [PANAS]
3. Feeling *worthless?* [PROMIS]
4. Feeling *excited?* [PANAS]
5. Feeling *that you had nothing to look forward to?* [PROMIS]
6. Feeling *strong?* [PANAS]
7. Feeling *helpless?* [PROMIS]
8. Feeling *enthusiastic?* [PANAS]
9. Feeling *sad?* [PROMIS]
10. Feeling *proud?* [PANAS]
11. Feeling *like a failure?* [PROMIS]
12. Feeling *alert?* [PANAS]
13. Feeling *unhappy?* [PROMIS]
14. Feeling *inspired?* [PANAS]
15. Feeling *hopeless?* [PROMIS]
16. Feeling *determined?* [PANAS]
17. Feeling *distressed?* [PANAS]
18. Feeling *attentive?* [PANAS]
19. Feeling *upset?* [PANAS]
20. Feeling *active?* [PANAS]
21. Feeling *guilty?* [PANAS]
22. Feeling *scared?* [PANAS]
23. Feeling *hostile?* [PANAS]
24. Feeling *irritable?* [PANAS]
25. Feeling *ashamed?* [PANAS]
26. Feeling *nervous?* [PANAS]
27. Feeling *jittery?* [PANAS]
28. Feeling *afraid?* [PANAS]

**Focus Groups – EMA-specific questions**

“We’re going to switch gears to talking about ways you could measure mood. Here are some examples of how we can ask you questions related to your daily mood. It is an app that can be designed to be used as a mood tracker, and it can be accessed from a phone.”

**Show examples of EMAs**

**Q: How many times a day would you be able to take a minute to talk about mood? 5 times? More? Less? – Why is this a good number?**

**Show examples of response scales**


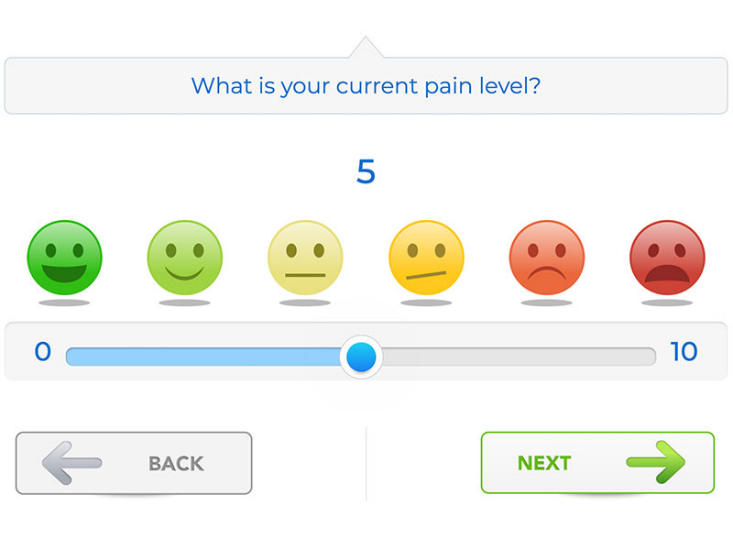


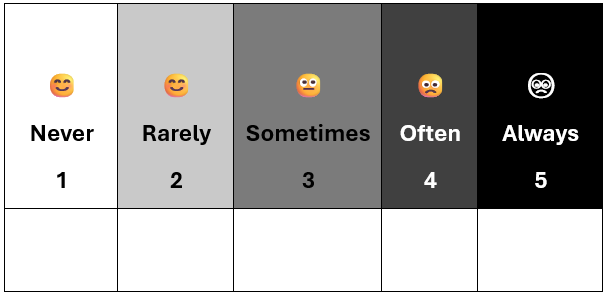


*
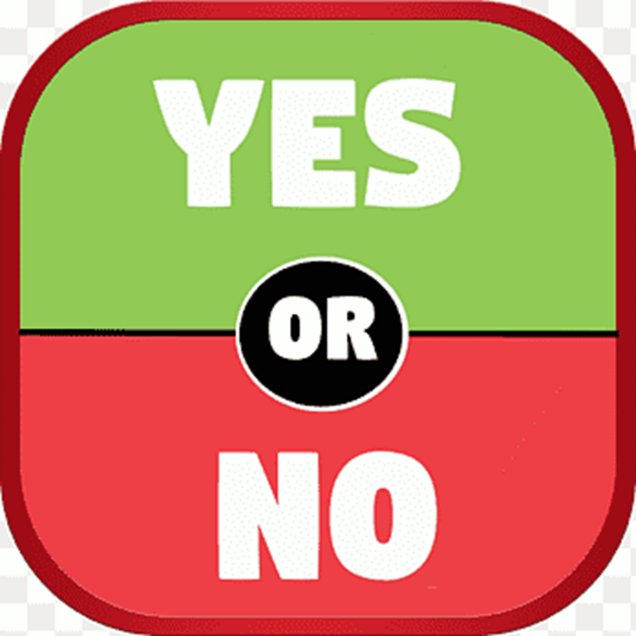
*

**Q: Tell me what you think of this format. Would you like this format? (Yes/No)**

**Q: What do you like about it? What do you not like about it? What would you change about it?**

**Q: Are there any other thoughts on the measures we’ve prepared?**

**Q: Are there any thoughts on your mental health in general that you want to share?**

**Cognitive Interview Guide**

**Item 1 – *Sad***

****show picture depicting [sad]****

Q: What emotion do you see in this picture [sad]?

Q: Do you think this picture shows SAD?

Q: What does SAD mean to you?

***show emotion scale***

Q: What does this [emotion scale] look like? What do the faces represent?

Q: If you were feeling SAD right now, how would you rate your mood on this scale?

Q: If you were NOT feeling sad right now, how would you rate your mood on this scale?

Q: What does RIGHT NOW mean to you?

Q: What were you thinking about when you answered this question?

Q: Was this question easy, medium, or hard to understand?

Q: Were you able to find the answer you wanted from these choices?

Q: What time period were you thinking about when you answered this question?

*Provide options: This week? Last week? Earlier today? Right now?*

**Item 2 – *Proud***

****show picture depicting [proud]****

Q: What emotion do you see in this picture [proud]?

Q: Do you think this picture shows PROUD?

Q: What does PROUD mean to you?

Q: If you were feeling PROUD right now, how would you rate your mood on this scale?

Q: If you were NOT feeling PROUD right now, how would you rate your mood on this scale?

Q: What were you thinking about when you answered this question?

Q: Was this question easy, medium, or hard to understand?

Q: Were you able to find the answer you wanted from these choices?

Q: What time period were you thinking about when you answered this question?

*Provide options: This week? Last week? Earlier today? Right now?*

**Item 3 – *Determined***

****show picture depicting [determined]****

Q: What emotion do you see in this picture [Determined]?

Q: Do you think this picture shows DETERMINED?

Q: What does DETERMINED mean to you?

Q: If you were feeling DETERMINED right now, how would you rate your mood on this scale?

Q: If you were NOT feeling DETERMINED right now, how would you rate your mood on this scale?

Q: What were you thinking about when you answered this question?

Q: Was this question easy, medium, or hard to understand?

Q: Were you able to find the answer you wanted from these choices?

Q: What time period were you thinking about when you answered this question?

*Provide options: This week? Last week? Earlier today? Right now?*

**Item 4 – *Like a Failure***

****show picture depicting [like a failure]****

Q: What emotion do you see in this picture [like a failure]?

Q: Do you think this picture shows LIKE A FAILURE?

Q: What does LIKE A FAILURE mean to you?

Q: If you were feeling LIKE A FAILURE right now, how would you rate your mood on this scale?

Q: If you were NOT feeling LIKE A FAILURE right now, how would you rate your mood on this scale?

Q: What were you thinking about when you answered this question?

Q: Was this question easy, medium, or hard to understand?

Q: Were you able to find the answer you wanted from these choices?

Q: What time period were you thinking about when you answered this question?

*Provide options: This week? Last week? Earlier today? Right now?*

**Item 5 – *Interested***

****show picture depicting [interested]****

Q: What emotion do you see in this picture [interested]?

Q: Do you think this picture shows INTERESTED?

Q: What does INTERESTED mean to you?

Q: If you were feeling INTERESTED right now, how would you rate your mood on this scale?

Q: If you were NOT feeling INTERESTED right now, how would you rate your mood on this scale?

Q: What were you thinking about when you answered this question?

Q: Was this question easy, medium, or hard to understand?

Q: Were you able to find the answer you wanted from these choices?

Q: What time period were you thinking about when you answered this question?

*Provide options: This week? Last week? Earlier today? Right now?*
