# Supplementary material for: Measuring affective symptoms of depression in aphasia: development of an accessible ecological momentary assessment tool
Source: Qual Life Res. 2026 Jun 6;35(7):175. doi: 10.1007/s11136-026-04292-y (PMC13242487; doi:10.1007/s11136-026-04292-y)
Supplement: Supplementary file 2 — Supplementary Material 2 [file 11136_2026_4292_MOESM2_ESM.docx]

**COREQ (COnsolidated criteria for REporting Qualitative research) Checklist**

| **Topic** | **Item No.** | **Guide Questions/Description** | **Reported on Page No.** |
| --- | --- | --- | --- |
| **Domain 1: Research team and reflexivity** | | | |
| *Personal Characteristics* |  |  |  |
| Interviewer/facilitator | 1 | Which author/s conducted the interview or focus group? | Title page & 4 |
| Credentials | 2 | What were the researcher’s credentials? | 3 |
| Occupation | 3 | What was their occupation at the time of the study? | 3 |
| Gender | 4 | Was the researcher male or female? | N/A |
| Experience and training | 5 | What experience or training did the researcher have? | 2 |
| *Relationship with participants* |  |  |  |
| Relationship established | 6 | Was a relationship established prior to study commencement? | 2 |
| Participant knowledge of the interviewer | 7 | What did the participants know about the researcher? E.g., personal goals, reasons for doing the research | 2 |
| Interviewer characteristics | 8 | What characteristics were reported about the interviewer/facilitator? E.g., bias, assumptions, interests in the topic | 2 |
| **Domain 2: Study design** | | | |
| *Theoretical framework* |  |  |  |
| Methodological orientation and Theory | 9 | What methodological orientation was stated to underpin the study? | 2,3 |
| *Participant selection* |  |  |  |
| Sampling | 10 | How were participants selected? | 2 |
| Method of approach | 11 | How were participants approached? | 2 |
| Sample size | 12 | How many participants were in the study? | 2 |
| Non-participation | 13 | How many people refused to participate or dropped out? Reasons? | N/A |
| *Setting* |  |  |  |
| Setting of data collection | 14 | Where was the data collected? | 3 |
| Presence of non-participants | 15 | Was anyone else present besides the participants and researchers? | 3 |
| Description of sample | 16 | What are the important characteristics of the sample? | 2, 3 |
| *Data collection* |  |  |  |
| Interview guide | 17 | Were questions, prompts, guides provided by the authors? Was it pilot tested? | 3, 4, supplementary materials |
| Repeat interviews | 18 | Were repeat interviews carried out? If yes, how many | 2 |
| Audio/visual recording | 19 | Did the research use audio or visual recording to collect the data? | 5 |
| Field notes | 20 | Were field notes made after the interview and/or focus group? | 5, 6 |
| Duration | 21 | What was the duration of the interviews or focus group? | 6 |
| Data saturation | 22 | Was data saturation discussed? | N/A |
| Transcripts returned | 23 | Were transcripts returned to participants for comment and/or correction? | N/A |
| **Domain 3: analysis and findings** | | | |
| *Data analysis* |  |  |  |
| Number of data coders | 24 | How many data coders coded the data? | 5, 6 |
| Description of the coding tree | 25 | Did authors provide a description of the coding tree? | N/A |
| Derivation of themes | 26 | Were themes identified in advance or derived from the data? | N/A |
| Software | 27 | What software, if applicable, was used to manage the data? | 5, 6 |
| Participant checking | 28 | Did participants provide feedback on the findings? | N/A |
| *Reporting* |  |  |  |
| Quotations presented | 29 | Were participant quotations presented to illustrate the themes/findings? Was each quotation identified? | N/A |
| Data and findings consistent | 30 | Was there consistency between the data presented and the findings? | 6, 7, 8, 9, 10 |
| Clarity of major themes | 31 | Were major themes clearly presented in the findings? | 6, 7, 8, 9, 10 |
| Clarity of minor themes | 32 | Is there a description of diverse cases or discussion of minor themes? | N/A |

Developed from: Tong A, Sainsbury P, Craig J. Consolidated criteria for reporting qualitative research (COREQ): a 32-item checklist for interviews and focus groups. *International Journal for Quality in Health Care*. 2007. Volume 19, Number 6: pp. 349 – 357
